# ﻿Two new species of the *Longitarsusviolentus* group from China (Coleoptera, Chrysomelidae, Galerucinae, Alticini)

**DOI:** 10.3897/zookeys.1181.110538

**Published:** 2023-10-03

**Authors:** Zulong Liang, Alexander S. Konstantinov, Yongying Ruan, Zhiqiang Li, Zhengzhong Huang, Siqin Ge

**Affiliations:** 1 Institute of Zoology, Chinese Academy of Sciences, Beijing 100101, China Institute of Zoology, Chinese Academy of Sciences Beijing China; 2 University of Chinese Academy of Sciences, Beijing, 100101, China University of Chinese Academy of Sciences Beijing China; 3 Systematic Entomology Laboratory, USDA, ARS, c/o Smithsonian Institution, National Museum of Natural History, Washington, DC, USA Smithsonian Institution, National Museum of Natural History Washington United States of America; 4 Plant Protection Research Center, Shenzhen Polytechnic University, Shenzhen, Guangdong 518055, China Shenzhen Polytechnic University Shenzhen China; 5 Institute of Zoology, Guangdong Academy of Sciences, Guangdong Key Laboratory of Animal Conservation and Resource Utilization, Guangdong Public Laboratory of Wild Animal Conservation and Utilization, Guangzhou, Guangdong 510260, China Institute of Zoology, Guangdong Academy of Sciences Guangzhou China

**Keywords:** Beijing, flea beetles, Palearctic, taxonomy, Xinjiang

## Abstract

Two new species of *Longitarsus* Latreille, 1829 from China are described: *L.pekingensis* Liang, Konstantinov & Ge, **sp. nov.** (Beijing) and *L.xinjiangensis* Liang, Konstantinov & Ge, **sp. nov.** (Xinjiang). Images of dorsal and lateral habitus, pronotum, head, and male and female genitalia are provided. The records of *Longitarsusviolentus* Weise, 1893 and *Longitarsusweisei* Guillebeau, 1895 in China are discussed. Holotypes of *L.marguzoricus* Konstantinov in Konstantinov & Lopatin, 2000 and *L.violentoides* Konstantinov in Konstantinov & Lopatin, 2000 are illustrated with images of pronotum and median lobe of aedeagus. A key to species of *L.violentus* species group is provided.

## ﻿Introduction

*Longitarsus* Latreille, 1829 is the most speciose genus of flea beetles and are widespread on all continents, except Antarctica, with more than 700 species worldwide (Konstantinov and Lopatin 2000); of these, 354 species occur in the Palearctic Region (Döberl 2010, 2011; Cox 2015; Takizawa 2015). Most species of *Longitarsus* are monophagous or oligophagous, feeding on various species of angiosperms, with larvae feeding on roots and adults on leaves of their host plants (Windig 1991). Salvi et al. (2019) found a strict association between most of the closely related species and specific plant families, indicating a phylogenetically conserved host-plant association in *Longitarsus* species.

Systematic studies of Chinese *Longitarsus* started relatively late. Although the first species occurring in China were described in the late 19^th^ century (e.g. Baly 1876), Sicien Chen was the first to focus on Chinese leaf beetle fauna and published a series of works in the 1930s (Chen 1933, 1934a, b, c, 1939a, b, 1941), mainly on south-western China and neighbouring areas. He described more than 30 *Longitarsus* species during his lifetime and provided the first key to Chinese species of the genus (Chen 1939b), which has been updated by Gressitt and Kimoto (1963). Subsequently, Warchalowski (1970) reviewed the Chinese species of *Longitarsus*, providing the most complete taxonomic and faunistic data to date. Wang (1992) later studied the *Longitarsus* fauna of Hengduan Mountains and described seven more species. To date, 71 species have been recorded from China (Warchalowski 1970; Gruev and Merkl 1992; Yang et al. 2016).

**Figure 1.**
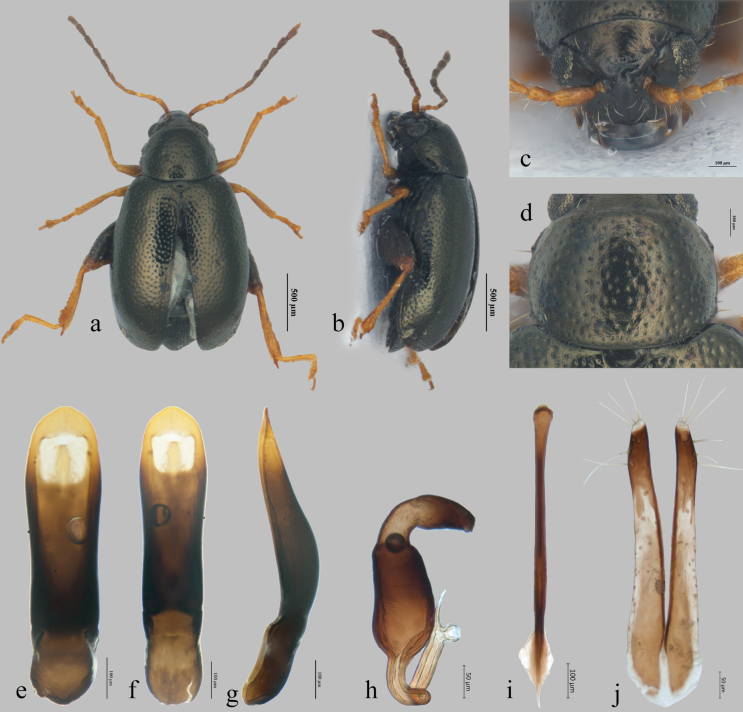
*Longitarsuspekingensis* Liang, Konstantinov & Ge, sp. nov. **a, b** habitus (**a** dorsal view **b** lateral view) **c** head **d** pronotum **e–g** median lobe of aedeagus (**e** ventral view **f** dorsal view **g** lateral view) **h** spermatheca **i** tignum **j** vaginal palpi.

**Figure 2.**
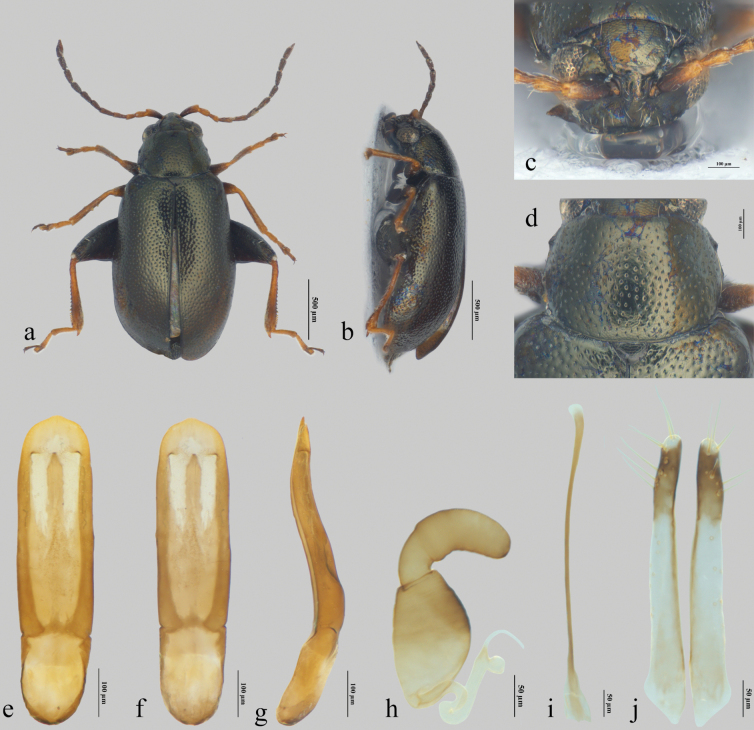
*Longitarsusxinjiangensis* Liang, Konstantinov & Ge, sp. nov. **a, b** habitus (**a** dorsal view **b** lateral view) **c** head **d** pronotum **e–g** median lobe of aedeagus (**e** ventral view **f** dorsal view **g** lateral view) **h** spermatheca **i** tignum **j** vaginal palpi.

As with many other species-rich flea beetle genera, *Longitarsus* may be divided into species groups. Some of them have been given a subgenus status (e.g. *Testergus* Weise, 1893) (Konstantinov 2005), while others have been informally treated (Leonardi and Doguet 1990; Biondi 1996). Konstantinov and Lopatin (2000) circumscribed a few southern Palearctic species which were assumed to present a species group around *L.asperifoliarum* Weise, 1887. This group was established based mainly on a combination of external morphological characters (see Konstantinov and Lopatin 2000). However, our subsequent (yet unpublished) studies show that these external features are widespread among *Longitarsus* species and unlikely to be diagnostic for species groups. Characters of male and female genitalia are better suited to recognize species groups in *Aphthona* Chevrolat, 1836 (Konstantinov and Lingafelter 2002), *Chaetocnema* Stephens, 1831 (Konstantinov and Lingafelter 2002; Ruan et al. 2019), and *Longitarsus* (Konstantinov 2005; Konstantinov and Dorr in press). Considering the variety of aedeagus morphologies, the *L.asperifoliarum* group seems artificial. Three species previously included in *L.asperifoliarum* group (i.e. *Longitarsusmarguzoricus* Konstantinov in Konstantinov and Lopatin 2000, *L.violentoides* Konstantinov in Konstantinov and Lopatin 2000, and *L.violentus* Weise, 1893) have similar structural plan of the median lobe of the aedeagus. Unlike other members of former *L.asperifoliarum* group, the median lobe of the aedeagus of these three species is S-shaped in lateral view (Figs 1g, 2g); in ventral view, it is relatively robust with wide, nearly parallel-sided ventral groove, and the apex is provided with wide, short to barely perceptible apical denticle (Figs 1e, f, 2e, f). The same general shape of the median lobe of aedeagus also is found in *L.pinguis* Weise, 1888 and *L.weisei* Guillebeau, 1895, which were omitted from Konstantinov and Lopatin’s (2000) study. Judging from the external morphology, as well as the morphology of aedeagus, these species mentioned above should be considered as a natural group, namely the *Longitarsusviolentus* group. Species of this group are characterized by the combination of the following characters: (1) the posterolateral callosities of the pronotum are poorly developed (low and wide), not visible from above; (2) dorsal surface, ventral surface, and metafemora are dark brown or black and with strong metallic blue, green, bronze, or brass reflection, and the reflection is weaker on ventral surface and metafemora; (3) the pro and mesofemora are dark brown or reddish brown at least basally; (4) the tibiae are light brown; (5) the vertex is strongly shagreened (except for the new species *L.pekingensis* described below); (6) pronotal surface is strongly shagreened (except for the new species *L.xinjiangensis* described below); (7) antennal calli are poorly developed, hardly delimited from vertex; (8) the frontal ridge is relatively wide, evenly convex, and not forming a sharp ridge; (9) the length of antennae is less than 2/3 of the body length; (10) the second antennomere is longer than the third, and as long as or slightly shorter than the fourth; (11) the metatibiae are straight in lateral view and slightly widening apically; (13) the median ridge of the metatibia is developed only in front of the apex; (14) median lobe of aedeagus is relatively robust and with deep ventral groove in ventral view, with apex provided with short to barely perceptible apical denticle; (15) the median lobe of the aedeagus is S-shaped in lateral view.

Two additional species clearly belonging to the *L.violentus* group were found in the insect collection of the Institute of Zoology, Chinese Academy of Sciences during our revisionary study of Chinese *Longitarsus*. One of them was later collected in Beijing. They are described below.

## ﻿Material and methods

Male and female of the new species were dissected, and genitalia were mounted in a drop of glycerol on slides for photographing. Images of the habitus and male genitalia were taken by using a Zeiss AXIO Zoom V16 microscope, and photographs of the female genitalia were taken by using a Zeiss AXIO Scope A1 microscope. The morphological terminology used in this study follows Konstantinov and Lopatin (2000). Labels written in Chinese are translated into English and cited verbatim.

Specimens studied in this paper are deposited in the following collections:

**IZCAS**Institute of Zoology, Chinese Academy of Sciences, Beijing, China;

**USNM**National Museum of Natural History [formerly, United States National Museum], Washington DC, USA.

## ﻿Results

### ﻿ Longitarsus pekingensis

Taxon classificationAnimaliaColeopteraChrysomelidae

Liang, Konstantinov & Ge sp. nov.

184B612C-C156-520A-9733-377E6B16EA30

https://zoobank.org/D7979324-63F8-4359-A031-A92BFAA7D00B

Figs 1a–j , 3a

#### Type materials.

***Holotype***: ♂ (IZCAS) China, Beijing, the China National Botanical Garden (North Section), Chinese rose garden, 80 m, 1 April 2023, host plant: *Bothriospermumchinense*, Dakang Zhou leg. ***Paratypes***: 1♂ 5♀ (IZCAS) same data as holotype; 1♀ (IZCAS) China, Beijing, the China National Botanical Garden (North Section), west of exhibition greenhouse, 80 m, 26 March 2023, host plant: *Bothriospermumchinense*, Dakang Zhou leg.; 3♀ (IZCAS) Beijing, Changping District, Liucun Town, Wangjiayuan, 20 October 2021, collected by net-sweeping, Meiying Lin leg.; 3♀ (IZCAS) Beijing, the Old Summer Palace, 21 November 1978, host plant: *Bothriospermumchinense*, Shuyong Wang leg.

#### Description.

Male body length 1.93–2.08 mm, width 0.95–1.07 mm; female body length 2.10–2.66 mm, width 1.02–1.32 mm. Body integument black with bronze reflection. Antennomeres I–V and legs yellowish to ferruginous, antennomeres VI–XI piceous. Metafemur black, dorsum with bronze reflection (Fig. 1a, b).

***Head*.** Vertex impunctate, with weak transverse wrinkles and well-developed supraorbital punctures near orbital sulcus. Antennal callus poorly developed, subtriangular, delimited from vertex by a shallow, barely perceptible groove, surface smooth (Fig. 1c). Orbital sulcus well developed, extending from the top of eye to the antennal socket. Midfrontal and suprafrontal sulci absent. Frontal ridge relatively wide and convex, distance between antennal sockets ca 2.4 times as wide as diameter of antennal socket. Anterofrontal ridge in middle as high as frontal ridge, slightly narrower than frontal ridge. Antennae short, length of antenna ca 0.62 times as long as body length, ratio of length of each antennomere 20: 13: 11: 13: 16: 13: 15: 14: 15: 16: 20. Antennomere II slightly longer than III, as long as IV.

***Thorax*.** Pronotum 1.33–1.37 times as wide as long. Lateral sides slightly convex, with maximum width in the middle. Anterolateral callosity well developed, slightly lower anteriorly, forming an acute angle. Lateral margin narrowly explanate. Posterolateral callosity low, slightly prominent. Punctures moderately large and dense, distance of interspace 1.5–2.1 times diameter of punctures. Interspaces weakly shagreened, stronger on pronotal disc (Fig. 1d). Scutellum semicircular, widely rounded apically, surface weakly shagreened. Elytra 3.14 times as long as pronotum, with well-developed humeral callus; maximum width in the middle. Apex broadly rounded. Punctures as large as punctures on pronotum but denser, width of interspaces 0.8–1.2 times as diameter of punctures. Interspaces weakly shagreened basally. Hind wings well developed.

***Legs*.** Metatarsomere I of male 0.54 times as long as metatibia, 1.23 times as long as metatarsomeres II–IV combined. Metatibial spur 0.63 time as long as width of metatibia (Fig. 1a). Tarsal claw simple, without denticle.

**Figure 3.**
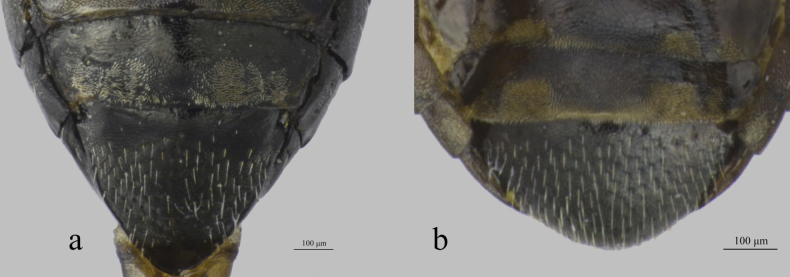
Last two tergites of female **a***Longitarsuspekingensis* Liang, Konstantinov & Ge, sp. nov. **b***Longitarsusxinjiangensis* Liang, Konstantinov & Ge, sp. nov.

***Abdomen*.** Preapical abdominal tergite of the female with distal area covered by short dense setae, extending forward to the middle at both sides but absent in the middle, sometimes middle area with few long setae. Apical abdominal tergite covered with long setae. A few minute microtrichia situated in the middle and lateral margins (Fig. 3a).

***Male genitalia*.** Median lobe of aedeagus slightly wider at about apical fourth, apex rounded, without denticle. Ventral side with wide groove which follows shape of entire lobe (Fig. 1e). Apical part dorsally with a subquadrate membranous window (Fig. 1f). In lateral view, the median lobe more or less straight; apical third of aedeagus gradually narrowed towards apex, dorsal side straight, ventral side convex (Fig. 1g).

***Female genitalia*.** Receptacle of spermatheca slender, 1.92 times as long as wide. Inner side of receptacle convex, outer side nearly straight. Basal part of pump rather long, about as long as apical part, well delineated from receptacle and from apical part of pump; apical part about as long as width of receptacle, narrowly rounded apically. Spermathecal duct forms two loops (Fig. 1h). Tignum slender, slightly curved, dilated apically (Fig. 1i). Vaginal palpus slender, posteriorly straight, narrowly rounded at apex (Fig. 1j).

#### Differential diagnosis.

*Longitarsuspekingensis* resembles *L.violentus*. It can be distinguished from the latter by the lack of sculpture on the vertex. Besides, the median lobe of aedeagus of *L.pekingensis* is more or less straight above basal opening in lateral view, without an apical denticle. Receptacle of spermatheca of *L.pekingensis* is more slender than that of *L.violentus*, and less convex on the inner side; the loops on the spermathecal duct are narrower in diameter. The key below allows to distinguish this species from all other species in the group.

#### Etymology.

The species is named after the type locality. The epithet is a noun in apposition.

#### Distribution.

Known from Changping District and Haidian District in Beijing, China.

#### Host plant.

*Bothriospermumchinense* Bunge (Boraginaceae).

### ﻿ Longitarsus xinjiangensis

Taxon classificationAnimaliaColeopteraChrysomelidae

Liang, Konstantinov & Ge sp. nov.

D9074AE3-50AC-5664-957A-74806BE4252C

https://zoobank.org/9D607213-F18E-445E-8EFC-7FD22BDADA51

Figs 2a–j , 3b

#### Type materials.

***Holotype***: ♂ (IZCAS) Xinjiang, Tianshan, Wukuerqi, 1120 m, 7 August 1957, Weiyi Yang leg.; ***Paratypes***: 4♀ (IZCAS) Tianshan, Wukuerqi, 1620 m, 7 August 1957; 2♂ (IZCAS) Xinjiang, Zhaosu, Wukuerqi, 1120–1620 m, 7 August 1957, Chunpei Hong leg.; 1♂ (IZCAS) Xinjiang, Zhaosu, Wukuerqi, 1120–1620 m, 7 August 1957, Guang Wang leg.; 1♂ (IZCAS) Xinjiang, Zhaosu, 1630 m, 11 August 1957, Chunpei Hong leg.; 1♂ 2♀ (IZCAS) Xinjiang, Burqin, 27 July 1955, Shijun Ma, Kailin Xia and Yonglin Chen leg.; 1♀ (IZCAS) Xinjiang, Burqin, 27 July 1955, Yonglin Chen et al. leg.; 1♂ (IZCAS) Xinjiang, Tacheng, 24 July 1955, Shijun Ma, Kailin Xia and Yonglin Chen leg.; 2♀ (IZCAS) Xinjiang, Urumqi, 14 July, 1956 Weiyi Yang leg.; 1♀ (IZCAS) Xinjiang, Urumqi, 20–890 m, 14 July 1959, Weiyi Yang leg.; 1♀ (IZCAS) Xinjiang, Xinyuan, 820–1200 m, 23 August 1957, Chunpei Hong leg.; 1♀ (IZCAS) Xinjiang, Xinyuan, 870–1220 m, 23 August 1957, Guang Wang leg.; 1♀ (IZCAS) Xinjiang, Tianshan, 2040 m, 13 August 1957, Guang Wang leg.; 1♂ (IZCAS) Xinjiang, Xiaotalate, 9 August 1956, Weiyi Yang leg.; 1♀ (IZCAS) Xinjiang, Jimsar, 14 May 1955, Shijun Ma and Yonglin Chen leg.; 1♀ (IZCAS) Xinjiang, Chaiwobao, 20 July 1956, Weiyi Yang leg.

#### Description.

Male body length 1.65–1.72 mm, width 0.78–0.90 mm; female body length 1.87–2.08 mm, width 0.89–1.18 mm. Body integument dark brown to black with bronze reflection. Apical part of antennomeres I–III yellowish to ferruginous, the rest part of antennae piceous. Legs ferruginous to piceous, metafemur black; dorsum with bronze reflection (Fig. 2a, b).

***Head*.** Vertex strongly shagreened, with well-developed supraorbital punctures near orbital sulcus. Antennal calli poorly developed, subtriangular, barely delimited from vertex, surface smooth (Fig. 2c). Orbital sulcus well developed, extending from top of eye to antennal socket. Midfrontal and suprafrontal sulci absent. Frontal ridge wide and convex. Distance between antennal sockets 2.28 times as great as diameter of antennal socket. Anterofrontal ridge in middle as high as frontal ridge, slightly narrower than frontal ridge. Antennae short, length of antenna ca 0.61 times as long as body length, ratio of length of each antennomere 20: 13: 11: 15: 16: 13: 16: 15: 16: 14: 20. Antennomere II longer than III, but shorter than IV.

***Thorax*.** Pronotum transverse, 1.44 times as wide as long. Lateral sides slightly convex, with maximum width at middle. Lateral margin of pronotum slightly angulate in front of middle (Fig. 2d). Anterolateral callosity well developed, slightly lower anteriorly, forming acute angle. Lateral margin narrowly explanate. Posterolateral callosity low, slightly prominent. Punctures large and dense, well defined, distance of interspace 0.9–1.7 times as diameter of punctures. Interspaces strongly shagreened, weaker in lateral area (Fig. 2d). Scutellum triangular, narrowly rounded apically, surface strongly shagreened. Elytra 3.86 times as long as pronotum, with well-developed humeral callus; maximum width behind middle. Apex broadly rounded. Punctures slightly larger and denser than those on pronotum, larger near suture, width of interspaces 0.9–1.1 times as diameter of punctures. Interspaces weakly shagreened basally. Hind wings well developed.

***Leg.*** Metatarsomere I of male 0.51 times as long as metatibia, 1.07 times as long as metarsomeres II–IV combined (Fig. 2a). Metatibial spur 0.66 times as long as width of metatibia. Tarsal claw simple, without denticle.

***Abdomen*.** Distal part of preapical abdominal tergite of female with two subquadrate areas on each side near apical part covered by short dense setae. Apical abdominal tergite covered with long setae. A few tiny microtrichia situated at middle (Fig. 3b).

***Male genitalia*.** Median lobe of aedeagus in ventral view with sides nearly parallel to each other, apex with poorly developed denticle. Ventral groove wide (Fig. 2e). Apical part dorsally with rectangular membranous window (Fig. 2f). Median lobe narrow in lateral view (Fig. 2g), curved above the basal opening, dorsal side and ventral side both convex apically.

***Female genitalia*.** Receptacle of spermatheca oval, 1.58 times as long as wide. Inner side of receptacle more convex than outer side. Basal part of pump rather long, about half as long as apical part, well delineated from receptacle and from apical part of pump; apical part longer than width of receptacle, widely rounded apically. Spermathecal duct forms two U-shaped turns (Fig. 2h). Tignum slender, slightly curved, dilated apically (Fig. 2i). Vaginal palpus slender, posteriorly straight, rounded at apex (Fig. 2j).

#### Differential diagnosis.

The key below allows for distinguishing *L.xinjiangensis* from all other species in the group. In addition, *L.xinjiangensis* is similar to *L.violentus* and *L.violentoides*. However, the elytra of *L.xinjiangensis* are slender, and the punctures on pronotum are larger. Compared to *L.violentus*, the antennomeres IV–VI of *L.xinjiangensis* are much darker, the median lobe of the aedeagus is broader, and the apex lacks an obvious denticle. Besides, the apex of the spermathecal pump of *L.xinjiangensis* is broader than that of *L.violentus*, and the spermathecal duct forms two U-shaped turns instead of loops. *Longitarsusxinjiangensis* can be distinguished from *L.violentoides* by the following characters: antennomere II shorter than IV; aedeagus narrow in lateral view; and apical part of spermathecal pump longer than width of receptacle.

#### Etymology.

The species is named after the type locality. The epithet is a noun in apposition.

**Figure 4.**
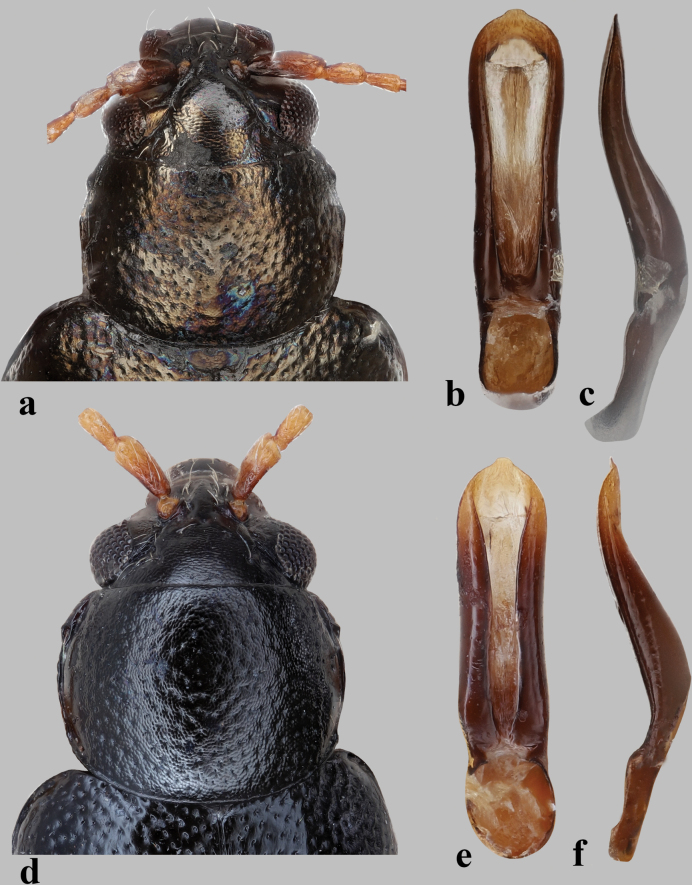
Pronotum and median lobe of aedeagus in *Longitarsus***a–c***L.marguzoricus*, holotype **a** pronotum **b, c** median lobe of aedeagus (**b** ventral and **c** lateral views) **d–f***L.pinguis***d** pronotum **e, f** median lobe of aedeagus (**e** ventral and **f** lateral views).

#### Distribution.

Known from northern Xinjiang, China.

**Figure 5.**
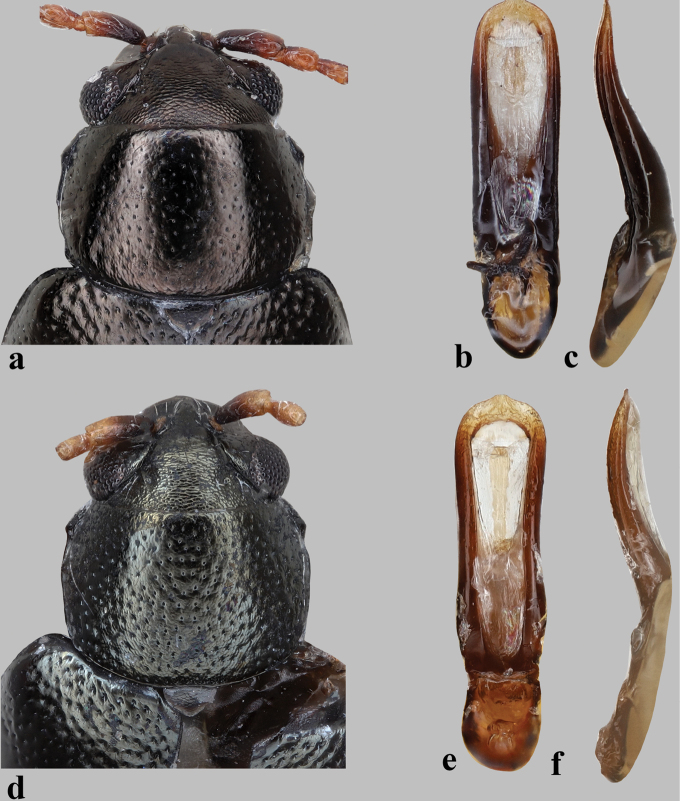
Pronotum and median lobe of aedeagus in *Longitarsus***a–c***L.violentoides*, holotype **a** pronotum **b, c** median lobe of aedeagus (**b** ventral and **c** lateral views) **d–f***L.violentus***d** pronotum **e, f** median lobe of aedeagus (**e** ventral and **f** lateral views).

#### Host plant.

Unknown.

**Figure 6.**
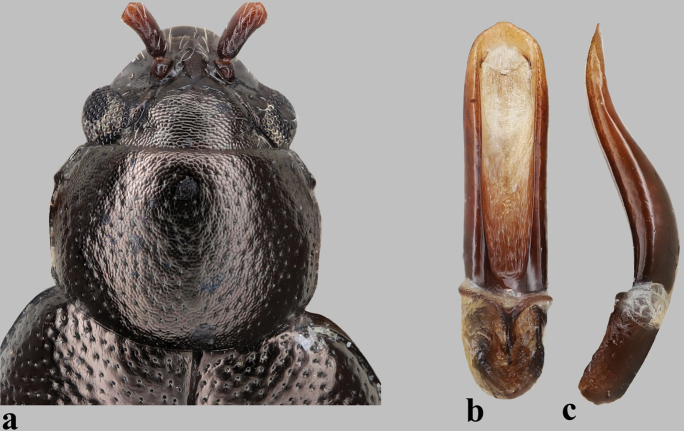
Pronotum and median lobe of aedeagus in *Longitarsusweisei***a** pronotum **b, c** median lobe of aedeagus (**b** ventral and **c** lateral views).

### ﻿Key to species of *Longitarsusviolentus* species group

**Table:** 

1	Vertex smooth, without perceptible sculpture (Fig. 1c); median lobe of aedeagus in lateral view more or less straight above basal opening (Fig. 1g)	***L.pekingensis* Liang, Konstantinov & Ge, sp. nov.**
–	Vertex strongly shagreened, or at least with transverse wrinkles (Figs 2c, 5d); median lobe of aedeagus in lateral view curved above basal opening (Fig. 5c)	**2**
2	Interspaces on pronotum strongly shagreened (Fig. 4d)	**3**
–	Interspaces on pronotum not strongly shagreened, smooth, or covered by wrinkles (Fig. 2d)	**6**
3	Basal part of antennomere I reddish brown (Fig. 4d); median lobe of aedeagus with ventral groove narrow, as narrow as the sclerotized lateral margins (Fig. 4e)	***L.pinguis* Weise**
–	Basal part of antennomere I piceous (Fig. 5a); median lobe of aedeagus with ventral groove wide, much wider than the sclerotized lateral margins (Fig. 5e)	**4**
4	Dorsal surface with a light purple tint (Fig. 6a); median lobe of aedeagus more or less parallel-sided medially in ventral view (Fig. 6b)	***L.weisei* Guillebeau**
–	Dorsal surface with a bronze or greenish tint (Figs 4a, 5d); median lobe of aedeagus more or less constricted medially in ventral view (Fig. 4b)	**5**
5	Last abdominal tergite of female with lateral microtrichia (see fig. 16 in Konstantinov and Lopatin 2000); median lobe of aedeagus slender, apical part less convex in lateral view (Fig. 5e)	***L.violentus* Weise**
–	Last abdominal tergite of female without lateral microtrichia (see figs 14, 15 in Konstantinov and Lopatin 2000). Median lobe of aedeagus more robust, apical part more convex in lateral view (Fig. 4b)	***L.marguzoricus* Konstantinov**
6	Pronotum with relatively large and densely placed punctures, distance of interspace 0.9–1.7 times as diameter of punctures (Fig. 2d)	***L.xinjiangensis* Liang, Konstantinov & Ge, sp. nov.**
–	Pronotum with relatively small and sparsely placed punctures, distance of interspace 2.1–3.8 times as diameter of punctures (Fig. 5a)	***L.violentoides* Konstantinov**

## ﻿Discussion

As currently understood *Longitarsusviolentus* group contains seven species. This number will undoubtedly increase when Central and Middle Asian faunas, as defined by Korotyaev et al. (2017), are properly reviewed. *Longitarsuslederi* Weise, 1889 has a median lobe of aedeagus somewhat similar to the *L.violentus* group as illustrated by Konstantinov (2005). *Longitarsuslederi* has been designated as the type species for the subgenus Testergus, and relationship of *L.violentus* group to *Testergus* needs to be further examined. Döberl (2010) in his “Catalogue of Palaearctic Coleoptera” reported the occurrence of *L.violentus* and *L.weisei* in China (in Fujian and Inner Mongolia, and Hebei, respectively). We could not track down the original literature records and could not find these species occuring in China. Overall, the presence of these species with the type localities in Europe and “Caucasus” in Kazakhstan, Altai, Inner Mongolia, Mongolia, Siberia, Far East, and Middle Asia is highly doubtful. Our data suggest that these records may be the results of misidentifications.

## Supplementary Material

XML Treatment for Longitarsus pekingensis

XML Treatment for Longitarsus xinjiangensis

## ﻿Additional information

### Author ORCIDs

Zulong Liang https://orcid.org/0000-0002-1289-2697

Alexander S. Konstantinov https://orcid.org/0000-0001-6578-6735

Yongying Ruan https://orcid.org/0000-0002-5025-5592

Zhengzhong Huang https://orcid.org/0000-0002-0085-6218

### Data availability

All of the data that support the findings of this study are available in the main text.
